# Little kidneys amid large global inequities

**DOI:** 10.1007/s00467-026-07147-3

**Published:** 2026-02-11

**Authors:** Judith Exantus, Maolynne Miller, Valerie A. Luyckx, Randall Lou-Meda

**Affiliations:** 1 Department of Pediatrics, Faculté de Médecine Et de Pharmacie, Université d’Etat d’Haïti, Port-Au-Prince, Haiti; 2 Service de Pédiatrie, Hôpital de L’Université d’Etat d’Haïti, Port-Au-Prince, Haiti; 3 Service de Pédiatrie, Hôpital Universitaire de Mirebalais, Mirebalais, Haiti; 4 Carvalho Kids, Kingston, Jamaica; 5 University Children’s Hospital, University of Zurich, Zurich, Switzerland; 6 Department of Public and Global Health, Epidemiology, Biostatistics and Prevention Institute, University of Zurich, Zurich, Switzerland; 7 Renal Division, Harvard Medical School, Brigham and Women’s Hospital, Boston, MA USA; 8 Department of Paediatrics and Child Health, University of Cape Town, Cape Town, South Africa; 9 European Kidney Health Alliance, Brussels, Belgium; 10 Programa de Salud Renal, Ministerio de Salud Pública y Asistencia Social, Guatemala City, Guatemala; 11 Guatemalan Foundation for Children with Kidney Diseases –FUNDANIER, Guatemala City, Guatemala

**Keywords:** Kidney disease, Inequity, Access to care, One health approach, Sustainable development goals, Social determinants of health

## Abstract

Chronic kidney disease (CKD) is a common non-communicable disease in children, and kidney dysfunction is the leading metabolic risk factor for death. Despite this, awareness of the CKD burden remains limited, and significant inequities exist in access to diagnosis and care worldwide. Kidney disease risk in children begins in utero and is dependent on the mother’s health and wellbeing. This is further impacted each day by poverty, nutrition, education, infection, and safety. Greater community awareness is needed, especially in lower resource settings, where children present late and may have no access to care. Early diagnosis, possibly supported by screening at schools, can have important public and individual health consequences. Catastrophic health expenditure is common if families attempt to pay out of pocket for kidney replacement therapy. Health systems require strengthening from the antenatal clinic through tertiary care to ensure children with kidney disease are identified and treated early, appropriately, affordably, and well. Local non-governmental organizations have had some success in mitigating inequities. Governments must step up, measure, and acknowledge the burden of kidney disease in children, ensure appropriate public health measures to reduce risk, strengthen primary care to improve the quality of diagnosis and care, and progressively scale up equitable access to all forms of kidney care. Kidney disease risk is strongly linked with social and structural determinants of health. A holistic approach to supporting child wellbeing—outlined by the Sustainable Development Goals and a One Health Approach—will positively impact child kidney health and promote equity among all children.

**Graphical abstract:**

**Figure:**
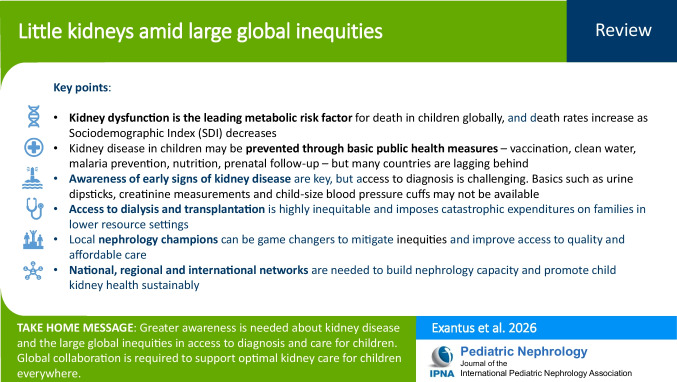
A higher resolution version of the Graphical abstract is available as Supplementary information

**Supplementary Information:**

The online version contains supplementary material available at 10.1007/s00467-026-07147-3.

Giving children a healthy start in life, no matter where they are born or the circumstances of their birth, is the moral obligation of every one of us.

Nelson Mandela, AIDS Vaccine Conference, South Africa, April 2002

## Global disparities in child and kidney health

Child mortality has declined since 2000, but progress has been uneven, and rates remain very high in many parts of Africa and southern Asia [1]. Disparities are greatest in young children, where the risk of death under age 5 varies 80-fold across the world [1]. In low-resource settings, the impact of non-communicable diseases [NCDs] on child mortality is growing [2]. Efforts to address NCDs have focused on the adult population, with children and the young largely being overlooked [3].

**Fig. 1:**
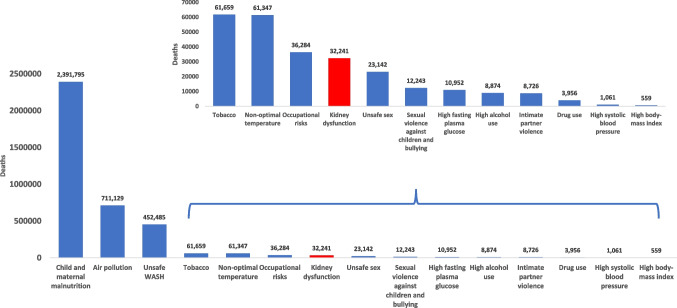
Global ranking of the leading risk factors for death in children in 2023. The main graph illustrates the number and ranking of the leading risk factors for death in children aged < 20 years globally. In the magnified inset, it can be seen that kidney dysfunction ranks as the 7th leading risk factor for death in children globally and is the leading metabolic risk factor for death, ranking above elevated blood glucose, high systolic blood pressure, and elevated body mass index. WASH – water, sanitation, hygiene. Data obtained from [5]

Chronic kidney disease (CKD) is one of the most common NCDs in children, with some estimates indicating that over 2 million children worldwide may have CKD stages 2–5 [4]. The 2023 Global Burden of Disease study suggests a prevalence of CKD stages 1–5 of 43 million in individuals under age 20 years and 12 million in individuals under age 14 years [5]. Although the burden has been less well described in children, these estimates are concerning because the Global Burden of Disease (GBD) study ranks kidney dysfunction as the leading metabolic risk factor for death in children, surpassing elevated fasting plasma glucose and high body mass index, which often attract more public attention [5, 6]. The same study also listed kidney dysfunction as the seventh most common risk factor for death in individuals under age 20 (Fig. 1, Supplementary Table 1). Despite this, awareness of the CKD burden in children and its importance remains limited, and most available data come from higher-income countries [4].

## Health system inequities impacting kidney health and kidney care

Early detection of kidney disease in children can provide significant public health benefits, but in settings where screening and diagnostics are unavailable or too costly, many children with kidney disease remain undiagnosed and likely die. Promoting kidney health requires collaboration across multiple sectors. Primary prevention focuses on addressing social and structural factors that impact health, especially in low- and middle-income countries (LMICs). Both primary and secondary prevention of kidney disease and its progression depend on early detection of risk factors or CKD itself, along with appropriate treatment and follow-up.

## Education and early diagnosis and care

Education must start in the community. Families and community health workers must be aware of simple measures, such as rehydration in acute kidney injury (AKI), avoidance of nephrotoxic remedies or medications, infection prevention strategies, and symptoms of kidney disease (like edema, urine appearance, failure to thrive), to avoid children being referred too late or not at all. Primary care clinicians must be aware of the need for early diagnosis of both AKI and CKD, to enable early and appropriate treatment to prevent progression of disease. Pediatric nephrologists can educate clinicians at all levels through conferences and outreach workshops in rural areas about when to suspect acute and chronic kidney disease, which types are more likely to progress, appropriate investigation of urinary tract infections, and clear indications for referral for pediatric nephrology consultation. Such efforts have resulted in earlier referrals and created opportunities for timely intervention [7, 8].

## Access to early diagnosis of kidney disease in children

### Antenatal screening

Sustaining a healthy pregnancy and delivery promotes fetal development and reduces the risk of neonatal and perinatal complications, including neonatal AKI [9]. The WHO recommends 8 antenatal clinic (ANC) visits [10], and that all pregnant women should undergo a prenatal ultrasound before 24 weeks of gestation to determine gestational age, parity, fetal well-being, and identify fetal anomalies [11]. Antenatal ultrasound is essential for early detection of congenital anomalies of the kidneys and urinary tract (CAKUT), helps anticipate potential delivery problems, guides management during pregnancy, and should prompt planning for referral and follow-up. Globally, ANC screening opportunities are being missed [10]. Especially in LMICs, common barriers include distance from services, transportation costs, study expenses, lack of trained ultrasonographers and equipment, and women’s educational levels and socioeconomic status [12, 13].

### Childhood screening

In LMICs, children with CKD often present with advanced disease and nonspecific symptoms, such as growth restriction, anemia, bone pain, and fatigue. Unless CKD is identified explicitly through blood and urine tests, the diagnosis is often missed [14, 15]. Access to diagnosis and treatment for kidney failure is associated with socioeconomic status and racial disparities [16–18].

Risk factors for both acute and chronic kidney disease include conditions present at birth, such as low birth weight (LBW) due to prematurity or being small for gestational age, CAKUT, and genetic disorders. Structural issues linked to poverty, malnutrition, systemic inequities, and environmental challenges also contribute [19]. In 2020, approximately 35.3 million babies were born too early or too small, representing 1 in 4 live births [20]. Most of these births occurred in LMICs, where screening and long-term follow-up are not routine. Consequently, these infants may face a lifelong risk of kidney disease, perpetuating health disparities.

**Fig. 2:**
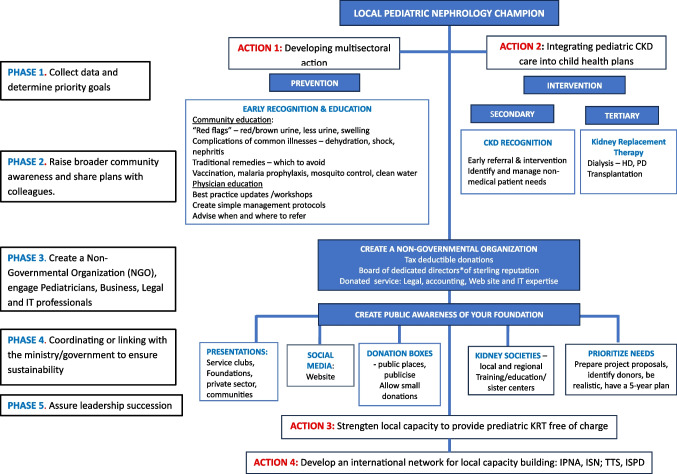
Practical steps towards mitigation of inequities and reducing the risk of kidney disease in children that can be initiated and led by local champions. Practical steps drawn from the author’s personal experiences, learned from key examples in Guatemala, Haiti, and Jamaica towards building a comprehensive plan built on multistakeholder and multisectoral action to improve kidney health and kidney care and mitigate inequities

Multiple settings offer opportunities for screening, as indicated in Fig. 2 and Supplementary Table 2 [9, 21–23]. Children with persistent abnormalities should be referred to pediatric nephrologists for further investigation. Although cost-effectiveness may be questioned, integrating screening into school medical evaluations could provide opportunities in settings where medical care encounters are infrequent [24]. Children at higher risk for CKD should be followed throughout their lifetimes, and targeted prevention strategies should be implemented. The application of these strategies is challenging due to the significant disparities and inequities that exist across the globe [25].

## Access to treatment for CKD

**Fig. 3:**
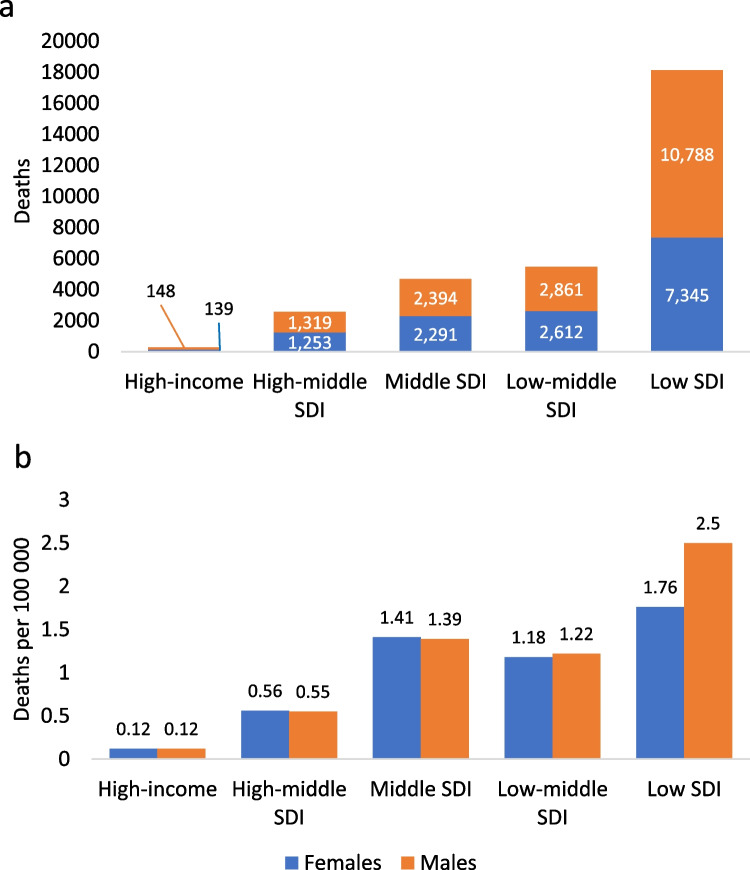
Deaths attributed to kidney dysfunction by sex and country sociodemographic index (SDI) in children < 20 years in 2023. The number of deaths (**a**) attributed to kidney dysfunction in children increases as country sociodemographic index (SDI) declines. The rates of death (**b**) attributed to kidney dysfunction overall are higher in boys, and are highest in boys in low SDI countries. This suggests that CAKUT, being disproportionately prevalent in boys, may be increasingly missed as country SDI declines or, alternatively, when family resources are limited, care may not be sought for girls in whom the diagnosis may be missed entirely. SDI is a measure of socioeconomic development encompassing years of schooling above age 15, lag-distributed income per capita, and fertility index under age 25 years. Data obtained from [5]

When stratified by a country’s sociodemographic index (SDI, a composite of per capita income, education level, and fertility rate) (Fig. 3), the death rates attributable to kidney dysfunction among children under age 20 increase as SDI decreases [6], reflecting differences in risk of kidney disease as well as access to timely diagnosis and appropriate treatment. Where poverty is rampant, or in areas where violence is commonplace, daily life is a struggle for survival, and early health care becomes a luxury until kidney failure can no longer be ignored.

One of the key sustainable development goal (SDG) targets for pediatric kidney care is achieving Universal Health Coverage (UHC), which is essential for promoting equity in healthcare access. Although UHC has increased, 3.8 billion people globally still lack coverage—mostly in LMICs, where coverage is 10%, compared to 86% in HICs [26]. The costs of transportation to medical facilities, as well as of diagnostic tests and treatment, may be prohibitive and impede adherence [27, 28]. Access to diagnostics may be challenging as often even basics such as urine dipsticks, creatinine measurements, and child-size blood pressure cuffs may not be available [29]. In many countries, even when medications for some chronic illnesses are covered, CKD-specific medications are often not included, putting heavy financial burdens on families with catastrophic health expenditures [30]. In government-supported hospitals and clinics, some costs may be covered, but it is not uncommon for patients to be asked to seek investigations and procure medication privately because of unavailability in the government facility [25, 31].

Access to pediatric nephrology is limited in many countries [29]. For example, in the Caribbean, the ratio of pediatric nephrologist/100,000 children varies from 0.56 in Jamaica to 0.08 in Haiti [personal communication]. In terms of resources, according to a recent survey, while most pediatric nephrology centers in LMIC were able to measure blood pressure and serum creatinine, perform urinalysis, urine culture and ultrasound, only about 82% were able to perform kidney biopsies [29]. Other supportive medical services such as pediatric surgery, radiology, and pathology were also inequitably available. Children with CKD often require surgery; however, for 1.5 billion children in LMICs, safe, affordable surgery remains inaccessible [32].

## Challenges with kidney replacement therapy in children

Kidney replacement therapy (KRT) availability increases as national income rises [33]. Even within HICs, however, despite having access to KRT, socio-economic circumstances impact outcomes in children requiring KRT [16, 18]. In LMICs, access is often limited by availability, geography, and affordability. Without government support for KRT, parents must pay out of pocket. The financial burden of treatment, medication, and transportation to the dialysis center—often far from home—may leave parents no choice but to decline KRT and accept death, or face financial ruin for the entire family [34–36].

In most LMICs, children who do access dialysis receive care in adult hemodialysis (HD) units, which often lack pediatric-specific HD supplies, and many are only offered treatment twice a week. Most children use HD catheters because, even though arteriovenous fistulas are the gold standard in chronic HD, the expertise for pediatric fistula creation is lacking in many LMICs [37, 38].

Although peritoneal dialysis (PD) is reportedly cheaper and more accessible than HD and can be performed at home, there are limitations because PD fluids are usually imported, expensive, and unpredictably supplied in LMICs. Additionally, the cost of Tenkohoff catheter placement could be prohibitive [39]. The Saving Young Lives Program has facilitated acute PD in many LMICs using improvised supplies, which has reduced mortality among treated children [40]. These compromises, however, are not feasible for long-term PD.

Kidney transplantation is recognized as the ideal treatment for childhood kidney failure. Worldwide, only 1 in 3 pediatric nephrology centers perform kidney transplants [29]. In LMICs, most transplants come from living related donors, so if there is no compatible relative, usually the mother, transplantation is unlikely. Cultural and religious factors, and a lack of local legislation, may hinder deceased donation [41]. A lack of knowledge about kidney transplantation and organ donation—whether living or deceased—contributes to the donor shortage.

## Kidney disease in children is a multiplier of inequities

### Impact of kidney disease in children on families and society

A CKD diagnosis in a child brings many changes to the child’s and the family’s lives. Caring for children with CKD can be stressful for families due to uncertainties, unpredictability, and necessary sacrifices, even leading to burnout, given their need to manage both parental and medical responsibilities [42]. Parental health literacy is associated with outcomes in children with CKD, and parents often feel they cannot provide the complex care required [43]. Catastrophic health expenses are a major concern [36]. Many parents lose their jobs because of the demands of the child’s treatment and face severe financial hardship due to medical and non-medical costs [34]. Indeed, the financial burden of caring for children with CKD is higher than that of other chronic illnesses, as they are hospitalized 12 times more often and incur higher medical costs [44].

Siblings of children with chronic illnesses often face neglect as parents concentrate more on the sick child [45]. They are at greater risk of developing anxiety, stress, sadness, depression, poorer cognitive development, or guilt over their own good health [46, 47]. Moreover, older siblings, especially sisters, are frequently tasked with caring for younger siblings with chronic illnesses in low-income or rural areas [48]. This can impact their education, social life, and overall well-being.

### Impact of kidney disease on child development

The growth and development of children and adolescents with CKD are hindered by disease complications, leading to inevitable lifelong increases in morbidity and mortality [49, 50]. Children with CKD are at risk for emotional, behavioral, and academic difficulties, as well as physical growth retardation [51]. Interrupting schooling may limit future educational and employment opportunities, which can further decrease socioeconomic status [49, 52]. In high-income settings, with support, children with CKD may achieve academic progress, although subsequent unemployment rates remain high [53]. Early detection and treatment of CKD and a holistic approach to care are essential to reduce these effects, improve quality of life, and optimize physical growth, cognitive function, and social-emotional well-being.

## Progress on the Sustainable Development Goals and the impact on child and kidney health

Although kidney disease in children is not a priority on the global health policy agenda, addressing many of the United Nations’ Sustainable Development Goals (SDGs) can reduce the risk of pediatric kidney disease [19]. For example, access to clean water (SDG 6), vaccination (SDG 3), and malaria prevention (SDG 3) can prevent infections, which are a common cause of AKI. AKI in children is predominantly community-acquired and often remains undiagnosed. Still, a recent meta-analysis of AKI in hospitalized children showed a mortality rate nearly three times higher in LMICs compared to HICs, likely reflecting the high severity of illness upon hospital presentation and/or lack of access to KRT [54, 55].

Achieving food security and improving nutrition (SDG 2) will reduce rates of both overnutrition and undernutrition [56]. Maternal undernutrition, very common in LMICs, is a well-known risk factor for infant LBW, an underappreciated risk factor for childhood CKD [9, 57]. Pediatric undernutrition increases the risk of infections and consequently AKI, while childhood obesity raises the risk of diabetes and accelerates CKD progression [58]. CKD in children, in turn, affects growth, worsens nutritional status, disrupts education (SDG 4), and limits future opportunities and socioeconomic status, perpetuating the cycle of poverty (SDG 1) [52]. SDG 5 and SDG 10, which focus on reducing gender and overall inequalities, are essential for improving maternal health and access to kidney care worldwide, as well as access to education and opportunities for girls [59].

**Fig. 4:**
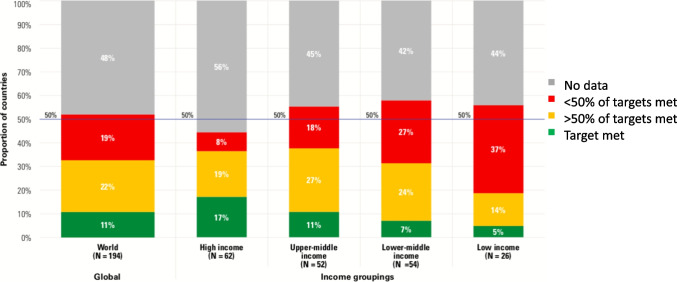
Global progress towards the child-related Sustainable Development Goals. The columns highlight world regions and the proportion of countries in 2023 that were on track to meet all of the child-related Sustainable Development Goals (SDGs) targets by 2030. The proportion of countries on track to meet the SDG targets declines with country income category (green bars). Almost half of countries are not reporting their progress (grey bars). Reproduced with permission from [60]

The setting of the SDGs encouraged countries (and donors) to make changes and track progress. By 2023, however, only 11% of countries were on track to fully achieve all the child-related SDG targets (Fig. 4) [60]. Alarmingly, data were missing from 48% of countries. Progress varies by income level, and if current trends continue, it is estimated that by 2030, only 1 in 4 children will live in a country where 70% of the SDG targets will have been achieved [26, 60].

SDG 16 emphasizes Peace, Justice, and Strong Institutions. This is especially important for children’s kidney health, which is increasingly affected by natural and man-made disasters [61]. During crises and conflicts, children’s kidney health can suffer in many ways, including increased gender-based violence, poor maternal health, higher risks of preterm birth and LBW, malnutrition, infections, injuries, mental health issues, and disrupted access to healthcare. The impact of these crises on child kidney health depends on the resilience of the health system. For example, in Turkey, where the health system is well prepared for earthquakes, the recent response to managing crush injuries in children was very successful [62]. In other settings, however, children’s kidney care needs are often neglected [61].

**Fig. 5:**
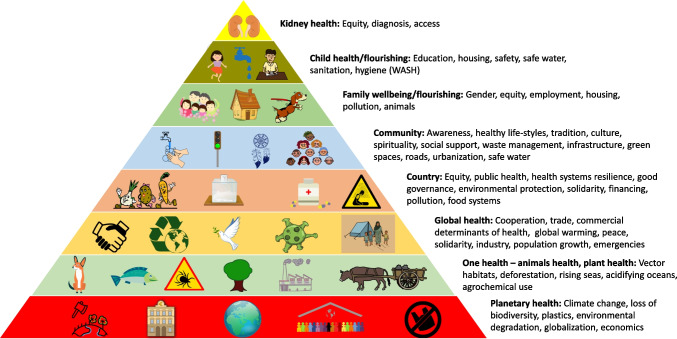
One Health approach to promote child kidney health. Child health, and child kidney health, is intimately related to the social and structural determinants of health. These determinants in turn are related to community, socioeconomic, environmental, and geopolitical factors which are all interlinked under the concepts of One Health and Planetary Health. Global solidarity is required to protect children and their kidneys. Images used obtained from Pixabay.com

Although not explicitly included in the SDGs, the One Health paradigm is highly relevant to child health. It links animal health, the environment, climate change, the food cycle, antimicrobial resistance, economics, geopolitics, and global governance, all of which influence equity, human health, and planetary well-being [63]. As depicted in Fig. 5, this paradigm also strongly relates to child kidney health. Adopting a One Health approach could significantly reduce disparities, strengthen health systems, and enhance well-being worldwide.

## Mitigating inequities in child kidney health—examples from Guatemala, Jamaica, and Haiti

In 2019, in the Region of the Americas, NCDs accounted for 30% of total deaths between ages 0–24 years, with percentages as high as 49.3% in the 5–9 year age group and 44.8% in the 10–14 year age group [64]. Remarkably, CKD is not mentioned in the Pan American Health Organization (PAHO)’s “Policy on Prevention and Control of Noncommunicable diseases in children, adolescents, and young adults” [3], despite kidney disease being among the top five causes of death from NCDs in people up to age 24 years (along with congenital anomalies, drug use disorders, brain and nervous system cancer) [64]. In 2021, Haiti, Guatemala, and Jamaica ranked 1st, 4th, and 25th, respectively, in terms of NCD deaths and 6th, 3rd, and 11th, respectively, in terms of leading CKD deaths [65, 66]. Despite these countries being within the Latin American and Caribbean Region, the experience of each in the provision of KRT is quite different. Pediatric KRT is not currently government-supported in either Jamaica or Haiti. Moreover, in Haiti, due to recurrent violence, the majority of children with CKD have been lost to follow-up (displaced families, closure of 2 university hospitals). The mortality rate from CKD is increasing because of dialysis unavailability at the closed facilities. In these countries, when government efforts fall short, local non-governmental organizations (NGOs), initiated by individual pediatric nephrologists, like the Jamaica Kidney Kids Foundation (JKKF) and the Guatemalan Foundation for Children with Kidney Diseases (FUNDANIER), have played a crucial role in building local capacity and supporting patients and families in LMICs [7, 8, 24, 67]. Although these NGOs have saved and improved the lives of local children and families, they tend to be built around a “local champion” of pediatric CKD, which limits their long-term sustainability unless they succeed in involving the countries’ institutions and fostering leadership succession [68, 69].

The Guatemalan government eventually invested in FUNDANIER, and with international support (including the International Pediatric Nephrology Association (IPNA), the International Society of Nephrology (ISN), and La Asociación Latinoamericana de Nefrología Pediátrica (ALANEPE), the program has evolved into a world-class pediatric nephrology program providing free pediatric KRT [30, 70]. In Jamaica**,** however, the government still has not invested in pediatric KRT, which, for children under age 12, is only offered in the fee-paying University Hospital of the West Indies (UHWI). JKKF, funded by donations from local and international, public and private sector sponsors, in partnership with the UHWI, provides pediatric-specific dialysis consumables in exchange for free dialysis. JKKF also subsidizes the cost of HD catheters, medications, investigations, and surgeries (e.g., fistula creation). While JKKF has prevented kidney failure deaths, its funding cannot support kidney transplantation or the development of local pediatric nephrology training [8]. Government support is needed to create the best service for Jamaican “kidney kids”. In Haiti, there is no “rescue NGO”, but the pediatric nephrologists there are “local champions” advocating for their patients through education and are tireless in their quest for support from private donors [71]. As shown in Guatemala, Jamaica, and Haiti, and as illustrated in Fig. 2, kidney care for children can be improved with simple measures, even when resources are minimal**.**

Looking back, it is clear that the activities carried out by these local NGOs have followed several common strategies: (1) Developing multisectoral actions to promote health and reduce pediatric CKD risk factors, with a focus on community education through health fairs, church talks, vaccine clinics, and schools [72, 73]; (2) Integrating pediatric CKD care into children’s health programs, achieved by educating healthcare professionals through courses on the most common pediatric nephrology diagnoses in collaboration with pediatric societies (like the Haitian Pediatrics Society), lectures at university pediatric units, and clinical rotations for residents and students; (3) Strengthening local capacity to provide comprehensive pediatric KRT free of charge since it is costly and only marginally available for children. Support includes providing dialysis catheters, subsidized medications, laboratory and radiological studies, as well as surgeries [7, 72]; (4) Developing an international network for local capacity building, which involves support from organizations like IPNA, ISN, and ALANEPE. This support includes training the pediatric nephrology workforce through courses [71], as well as conducting medical missions focused on building local capacity by training providers and planning follow-up activities [70]. Other successful models have emerged around the globe, such as the kidney care model in Nicaragua [74] and Pakistan, all of which attest to the strong value of international and local collaboration, mentorship, building local capacity and expertise, and a commitment to quality care [34, 74–76]. Key to the success of many of these programs is the enduring impact of fellowship training programs in pediatric nephrology which are serving to multiply a competent and dedicated pediatric nephrology work force and grow a global network that will only strengthen pediatric kidney care over time [77]. As adult nephrology services may be the only service available in many low-resource settings, a first step in strengthening and growing pediatric nephrology services may be to build capacity among the adult nephrology care team to manage children together with their pediatric colleagues [37, 78].

## Call to action

On May 23, 2025, the World Health Assembly adopted the first-ever resolution on kidney health [79]. This was long-awaited by the kidney community, showing that all countries now recognize kidney disease as a crucial part of the global NCD burden. The resolution urges countries to monitor the prevalence of kidney disease to better understand its global distribution and identify prevention and care needs. It also calls for improved prevention through public health measures, early recognition and treatment of risk factors, and strengthening health systems to enhance access to diagnosis and care at all levels. Because of the high costs of kidney care, the resolution advocates for support for health technology assessments, priority setting, and financing strategies to gradually expand access for everyone. Consequently, this places significant demands on countries and global institutions. Professional education is also key to addressing the underdiagnosis and underestimation of pediatric kidney disease in both clinical practice and public health policy. While this is necessary, we must remember that individuals can also make a difference. Starting an NGO when a need is identified can unite support and raise awareness within communities. In clinical settings, we can promote well-being at every stage of kidney disease by providing the best possible care and ensuring our environments support progress. We can also adopt a holistic approach to help children and their families, aiming to reduce hardship and foster well-being. The global disparities in access to and quality of care for children highlight that good care is achievable and that children with kidney disease can thrive. This must become a reality for all children everywhere.

## Supplementary Information

Below is the link to the electronic supplementary material.

Graphical abstract (PPTX 123 KB)

ESM 1 Supplementary Material 1 (DOCX 41.6 KB)
