# Supplementary material for: Little kidneys amid large global inequities
Source: Pediatr Nephrol. 2026 Feb 11;41(10):3197–208. doi: 10.1007/s00467-026-07147-3 (PMC13481463; doi:10.1007/s00467-026-07147-3)
Supplement: Supplementary file 1 [file 467_2026_7147_MOESM2_ESM.docx]

**Supplementary Table 1: Leading risk factors for death and potential impact of the Sustainable Development Goals on kidney health in children[1]**

| Rank | Leading risk factors for death age < 20 years | SDG | Population affected - examples | Impact on kidney health |
| --- | --- | --- | --- | --- |
| 1 | Child and maternal malnutrition | 1, 2, 4, 5 | 193 million children stunted or wasted in 2025 | Low birth weight, preterm birth -> low nephron number, long term risk CKD  Failure to thrive |
| 2 | Air pollution | 7, 9, 11, 12, 13 | Women and girls disproportionately exposed to indoor air pollution from unhealthy cooking fuels still used by 2.2 billion people | Low birth weight, preterm birth -> low nephron number, long term risk CKD |
| 3 | Unsafe water, sanitation, and handwashing (WASH) | 1, 4, 6 | 646 million children lack basic hygiene services at school | Diarrhoea -> AKI; impetigo -> glomerulonephritis |
| 4 | Tobacco | 3 | 1.25 billion people use tobacco | Hypertension, CKD |
| 5 | Non-optimal temperature | 13, 11, 14, 15 | 2024 was the hottest year on record | High temperatures -> Kidney stones, UTIs |
| 6 | Occupational risks | 1, 4, 8 | 138 million children in child labour, 54 million engaged in hazardous work | Environmental exposures, CKDu |
| **7** | **Kidney dysfunction** | 3 | ≥ 2 million children affected with CKD stages 2-5 | CKD, kidney failure, cardiovascular disease |
| 8 | Unsafe sex | 3, 4, 5 | 1.4 million children live with HIV, 86% in sub-Saharan Africa | HIV, AKI and CKD, medication toxicity |
| 9 | High alcohol use | 3 | Global alcohol consumption per capita 5 litres in 2022 | Trauma, rhabdomyolysis, chronic liver disease, AKI, CKD |
| 10 | High fasting plasma glucose | 3 | >1.2 billion affected globally | Diabetes, CKD |
| 11 | Intimate partner violence | 5, 10, 11 | >60% of young men and women experience sexual violence by age 18 years | Infections, trauma, teenage pregnancies, preeclampsia, low birth weight, preterm birth, AKI |
| 12 | Drug use | 3 | Around 1% of global population, more in HICs | Infections, endocarditis, hypertension -> AKI, glomerulonephritis |
| 13 | High systolic blood pressure | 3 | 1.3 billion affected globally | CKD, cardiovascular disease |
| 14 | High body-mass index | 3 | 35,5 million children overweight in 2025 | Diabetes, hypertension, CKD |

[2-12]

**Supplementary Table 2. Suggested screening opportunities for kidney disease in children in low and middle income settings**

| Who to test | When to start | How often? | Where | How* |
| --- | --- | --- | --- | --- |
| Low birth weight, preterm, maternal preeclampsia, | At one month after birth | Annual | Pediatrician, primary care | BP, Urine ACR, creatinine/eGFR  Ultrasound  Height, weight tracking |
| AKI, History of herbal poisoning | 3 months after AKI | Annual | Pediatrician, primary care | BP, Urine ACR, creatinine/eGFR  Ultrasound (at the initial visit)  Height, weight tracking |
| Family history of kidney disease | Depending on familial risk | Annual | Pediatrician, primary care | BP, Urinalysis, creatinine/eGFR  (ultrasound as indicated)  Height, weight tracking |
| CAKUT,  Neural tube defect | After birth, as soon as identified | Annual or more frequently as needed | Nephrology, Urology | BP, Urinalysis, Urine ACR, creatinine/eGFR  Ultrasound, bladder evaluation (VCUG)  Height, weight tracking |
| Pyelonephritis – recurrent/atypical bacteria | After index episode | As required | Nephrology, Urology | Ultrasound, exclude reflux (VCUG)  BP, Urinalysis, Urine ACR, creatinine/eGFR  Height, weight tracking |
| Children in background high risk settings – IgA nephropathy, CKDu, chronic disease (cancer, cardiac disease, diabetes, hypertension, HIV/AIDS) | School age | Every 6 months or as needed | Pediatrician, Nephrology | BP, Urinalysis, Urine ACR, creatinine/eGFR,  Ultrasound (at least once)  Height, weight tracking |
| Children in background high risk settings – recurrent AKI, Malaria, sickle cell, use of herbal medication | School age | Annual or more frequently as needed | Pediatrician, primary care, school health checks | BP, Urinalysis for hematuria, creatinine/eGFR,  Ultrasound (at least once)  Height, weight tracking |
| Overweight | When detected | Annual | Pediatrician, primary care | BP, Urine ACR, creatinine/eGFR  Ultrasound (at least once)  Height, weight tracking |
| Urinary abnormalities, prolonged, bedwetting, polyuria | When detected | As required | Pediatrician, primary care | BP, Urinalysis, Urine ACR, creatinine/eGFR  Ultrasound (at least once)  Height, weight tracking |
| Growth failure | When detected | As required | Pediatrician, primary care | BP, Urinalysis, Urine ACR, creatinine/eGFR,  Ultrasound (at least once)  Height, weight tracking |
| Urolithiasis | When detected | Annually or more frequently as needed | Pediatrician, Nephrology | BP, Urine dipstick for proteinuria and hematuria,  creatinine/eGFR, metabolic panel in blood and urine, growth |
| Children with glomerular disease : Nephrotic syndrome, acute glomerulonephritis | When detected | As clinically indicated | Pediatrician, Nephrology | BP, urine dipstick for proteinuria and hematuria, Urine ACR, creatinine/eGFR, serum Albumin, growth |

Table input derived form references [13-18] and author opinion. ACR – albumin creatinine ratio, AKI – acute kidney injury, BP – blood pressure, CAKUT – congenital abnormalities of the urinary tract, CKDu – chronic kidney disease of unknown etiology, eGFR – estimated Glomerular Filtration rate, HIV/AIDS – human immunodeficiency virus/acquired immunodeficiency syndrome, VCUG – voiding cystourethrogram.

1.	Institute for Health Metrics and Evaluation (2025) GBD Results. Institute for Health Metrics and Evaluation, Seattle, Washington. <https://vizhub.healthdata.org/gbd-results/>

2.	FAO, IFAD, UNICEF, WFP, WHO. (2023. ) The State of Food Security and Nutrition in the World 2023. Urbanization, agrifood systems transformation and healthy diets across the rural–urban continuum. Food and Agricultural Organization of the United Nations (FAO), Rome. <https://doi.org/10.4060/cc3017en>

3.	UNICEF (2025) Child Malnutrition. UNICEF, New York. <https://data.unicef.org/topic/nutrition/malnutrition/>

4.	United Nations Children’s Fund (UNICEF), World Health Organization (WHO) (2024) Progress on Drinking Water, Sanitation and Hygiene in Schools 2015–2023: Special focus on menstrual health. . UNICEF and WHO, New York. <https://washdata.org/reports/jmp-2024-wash-schools>

5.	Nations. U (2025) The Sustainable Development Goals Report 2025. United Nations, New York. <https://unstats.un.org/sdgs/report/2025/>

6.	UNICEF. (2023) Progress on Children's Well-being: Centring child rights in the 2030 Agenda. For Every Child, A Sustainable Future. UNICEF Division of Data, Analytics, Planning and Monitoring – Data and Analytics Section, New York. <https://data.unicef.org/resources/sdg-report-2023/>

7.	Our World in Data (2023) Ensure access to affordable, reliable, sustainable and modern energy for all. Our World in Data, Oxford. <https://ourworldindata.org/sdgs/affordable-clean-energy>

8.	Institute for Health Metrics and Evaluation [IHME] (2024) GBD Compare Data Visualization. University of Washington, IHME, Seattle, WA. <http://vizhub.healthdata.org/gbd-compare>.

9.	Ritchie H, Arriagada P, Roser M (2022) Opioids, Cocaine, Cannabis, and Other Illicit Drugs. Our World in Data Oxford. <https://ourworldindata.org/illicit-drug-use>

10.	United Nations Children’s Fund (UNICEF), World Health Organization, Bank. TW (2025) Levels and trends in child malnutrition: UNICEF / WHO / World Bank Group Joint Child Malnutrition Estimates. Key findings of the 2025 edition. World Health Organization, Geneva

11.	World Health Organization (2023) Global report on hypertension: the race against a silent killer. World Health Organization, Geneva. <https://www.who.int/publications/i/item/9789240081062>

12.	International Diabetes Federation (2025) IDF Diabetes Atlas 2025. International Diabetes Federation, Brussels. <https://diabetesatlas.org/resources/idf-diabetes-atlas-2025/>

13.	Karmakar S, Dasgupta D, Akhtar S, Poddar S, Giri PP, Tse Y, Sinha R (2025) Progression of acute kidney injury to chronic kidney disease: a prospective cohort study. Pediatr Nephrol 40:3293-3300.doi:10.1007/s00467-025-06810-5

14.	Conroy AL, Opoka RO, Bangirana P, Idro R, Ssenkusu JM, Datta D, Hodges JS, Morgan C, John CC (2019) Acute kidney injury is associated with impaired cognition and chronic kidney disease in a prospective cohort of children with severe malaria. BMC Med 17:98.doi:10.1186/s12916-019-1332-7

15.	Kamath N, Nesargi S, Bajpai D, Singarayar P, Vidal E, Luyckx VA (2025) Impact of maternal health on neonatal and long-term kidney outcomes. Pediatr Nephrol.doi:10.1007/s00467-025-06771-9

16.	De Mul A, Parvex P, Wilhelm-Bals A, Saint-Faust M (2020) Renal follow-up in pediatrician practice after discharge from neonatology units: about a survey. European journal of pediatrics 179:1721-1727.doi:10.1007/s00431-020-03652-1

17.	Nelson-Piercy C, Srisawat N, Kashani K, Lumlertgul N, Murugan R, Rhee H, Chakravarthi R, Surapaneni T, Acharya A, Ankawi G, Bramham K, Cerda J, Clark K, Claure-Del Granado R, Gowrishankar S, Luyckx V, Menon S, Poli-de-Figueiredo CE, Ramachandran R, Sahay M, Samavedam S, Shemies R, Shetty MS, Wiles K, Wium L, Wu VC, Yadla M, Ronco C, Mehta RL, Ostermann M (2025) Pregnancy-associated acute kidney injury - consensus report of the 32nd Acute Disease Quality Initiative workgroup. Nat Rev Nephrol 21:633-646.doi:10.1038/s41581-025-00979-6

18.	Nogueira PCK, Konstantyner T, Carvalho MFC, Pinto CCX, Paz IP, Belangero VMS, Tavares MS, Garcia CD, Neto OAF, Zuntini K, Lordelo MDR, Oi SSP, Damasceno RT, Sesso R (2019) Development of a risk score for earlier diagnosis of chronic kidney disease in children. PloS one 14:e0215100.doi:10.1371/journal.pone.0215100
